# Asymmetric hydrosilylation of ketones catalyzed by complexes formed from *trans*-diaminocyclohexane-based diamines and diethylzinc

**DOI:** 10.1007/s00706-012-0754-0

**Published:** 2012-04-18

**Authors:** Jadwiga Gajewy, Jacek Gawronski, Marcin Kwit

**Affiliations:** Department of Chemistry, A. Mickiewicz University, 60-780 Poznan, Poland

**Keywords:** Homogeneous catalysis, Metal complexes, Ligands, Macrocycles, Asymmetric activation, Zinc

## Abstract

**Abstract:**

Chiral acyclic and macrocyclic amines derived from *trans*-1,2-diaminocyclohexane in complexes with diethylzinc efficiently catalyze asymmetric hydrosilylation of aryl–alkyl and aryl–aryl ketones with enantiomeric excess of the product up to 86 %. A trianglamine ligand with a cyclic structure or the presence of an additional coordinating group increases the enantioselectivity of the reaction, in comparison with catalysis by a simple acyclic *N*,*N*′-dibenzyl-1,2-diaminocyclohexane ligand. In addition, the effect of the asymmetric activation of the catalyst by a variety of alcohols and diols is studied.

**Graphical Abstract:**

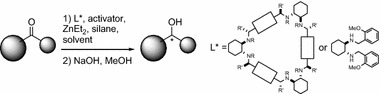

**Electronic supplementary material:**

The online version of this article (doi:10.1007/s00706-012-0754-0) contains supplementary material, which is available to authorized users.

## Introduction

**Scheme 1:**
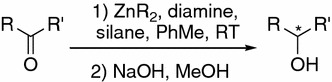

Secondary alcohols are an important class of chiral building blocks in organic synthesis and form structural fragments of numerous biologically active compounds [1]. Major asymmetric catalytic methods for preparation of secondary alcohols rely mainly either on asymmetric hydrogenation of ketones, with use of chiral rhodium complexes as catalysts in both homogeneous and heterogeneous approaches, or on oxazaborolidine-catalyzed asymmetric reduction of ketones with diborane [2–6]. Before the re-invention of the reductive properties of polymethylhydrosiloxane (PMHS), a safe and inexpensive by-product of the silicon industry, hydrosilylation of C=O bonds seemed a less convenient method because of the toxicity and cost of monomeric silanes. Since the discovery of Zn-diamine-catalyzed asymmetric hydrosilylation of prochiral ketones, several other methods for enantioselective reduction of the C=O bonds have been developed [7–23]. They are mainly based on the use of chiral transition metal complexes with P,P-bidentate ligands, P,S-ligands, N-ligands, or N-heterocyclic carbene ligands. Although the enantioselectivity obtained by use of these complexes is >90 %, use of such complexes suffers from high cost and elaborate preparation. In contrast, use of catalytic systems based on zinc has emerged as a promising and advantageous method for metal-catalyzed asymmetric hydrosilylation of prochiral ketones (Scheme 1).

According to the original procedure developed by Mimoun et al. [7] the reaction requires chiral 1,2-diamine as ligand for dialkylzinc. Modifications could be made either by judicious choice of chiral ligand, by adding an activator, or by changing the reaction conditions (temperature, solvent). The ligands were usually obtained from commercially available amines, for example 1,2-diamino-1,2-diphenylethane or 1-phenylethylamine. However, enantiomerically pure *trans*-1,2-diaminocyclohexane (DACH) is the preferred source of a virtually unlimited number of diamine structures. Apart from simple acyclic *N*,*N*′-disubstituted derivatives, the ability to make complex structures of triangular, rhombic, or spherical character from suitable substrates is well documented for DACH [24–29]. Another possibility of modifying the structure and properties of macrocyclic DACH derivatives is available by further *N*-substitution and/or by formation of additional chiral centers at the benzylic positions and introduction of other functional groups in the ligand structure [30, 31]. Chiral macrocyclic tetramine and hexamine macrocycles derived from *trans*-1,2-diaminocyclohexane in complexes with diethylzinc efficiently catalyze asymmetric hydrosilylation of aryl–alkyl ketones and imines with enantiomeric excess of the product up to 99 % [32, 33].

**Scheme 2:**
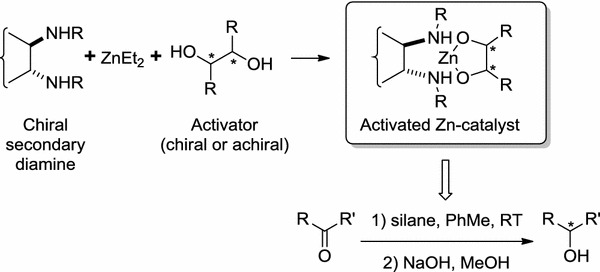

Introduction of two different chiral centers into the ligand structure may give rise to synergistic effects, leading to and increase in the enantioselectivity of hydrosilylation reaction [34]. The basic concept consists in the idea of “asymmetric activation”, introduced by Mikami and co-workers and subsequently applied to several enantioselective transformations [35–39]. By following this concept, Ushio and Mikami have developed an efficient method for hydrosilylation of *ortho*-substituted benzophenones by use of chiral [Zn(diamine)(diol)] complexes, with chirality residing in the diamine part of the complex (Scheme 2) [38]. Prochiral benzophenones can be enantioselectively reduced with up to 96 % ee of the product. The effect of an activator on the stereochemistry of the product was negligible, because enantioselectivity was comparable irrespective of whether (*R*) or (*S*)-BINOL, or ethylene glycol was used.

Recently Bette et al. [39, 40] reported Zn-promoted direct reduction of a variety of ketones to the corresponding alcohols with PMHS in protic solvents. Although the chemical yields of the reactions were quantitative, moderate enantioselectivity (ee up to 55 %) was obtained by use of a variety of enantiopure diamine ligands. Another possible means of improving the efficiency of the catalyst system was by changing the reaction conditions from an aprotic to a protic solvent—a rather uncommon situation in organometallic chemistry.

**Fig. 1:**
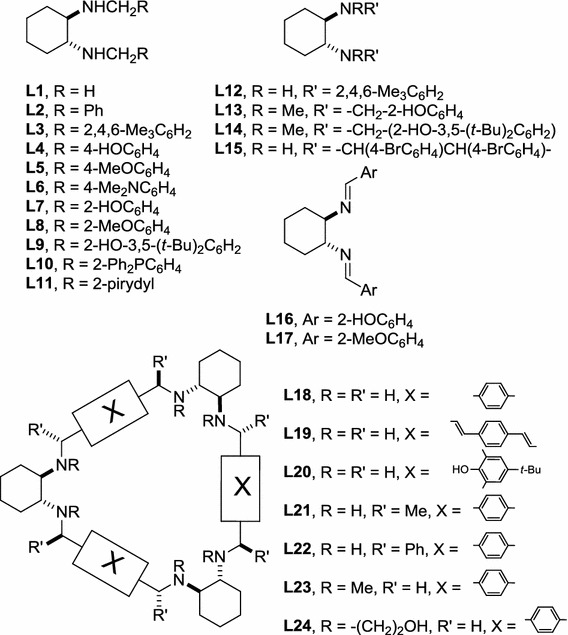
Acyclic and cyclic ligands used in this study

The main objective of this work was to test several acyclic or cyclic ligands derived from enantiomerically pure *trans*-1,2-diaminocyclohexane, which are secondary or tertiary diamines, imines, or heteraphanes with additional substituents with potential coordinating character (Fig. 1).

According to Mimoun’s model and our recent results, coordination of the ketone substrate to the ZnR_2_–amine complex results in release of an alkane (RH) molecule with the formation of Zn···O coordinated species in which one of the nitrogen atoms forms a bond to the carbon atom of the carbonyl group [7, 30]. Complexation of the ketone molecule with C–N bond formation is accompanied by significant changes of the conformation of the ligand around the coordination site. Alternatively, hydrosilylation may take place by direct silane attack on the activated carbonyl group without significant change of structure of the complex [30]. Ligands used in this study may act according to the first or second model.

This study was inspired by the work of Ushio and Mikami [38] and Costa et al. [37], who reported that enantiopure [Zn(diamine)] species could be activated by either enantiopure BINOL or achiral 1,2 or 1,3-diol ligands to form efficient catalysts for asymmetric hydrosilylation of ketones. We were particularly interested in the application of achiral or chiral diols as activators, because the number of available diols is higher than the number of chiral diamines and because the diamines are usually synthesized from the corresponding diols.

**Fig. 2:**
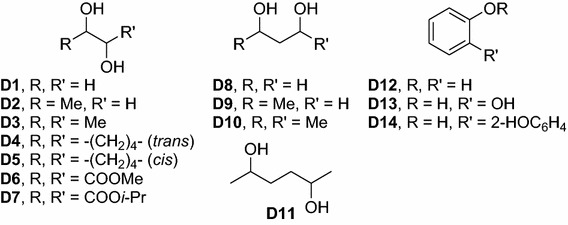
Diols and alcohols used as activators in [Zn(diamine)(diol)]-catalyzed hydrosilylations

We tested several diols (Fig. 2), either chiral or achiral, in combination with appropriate diamines. For chiral diols both enantiomers and racemic mixtures were used, if available.

The effects of temperature and the structure of the silane were also investigated. As test substrate for the [Zn(diamine)(diol)]-catalyzed reactions we selected 4-methylacetophenone; the general reaction conditions were as shown in Scheme 1, where R = 4-MeC_6_H_4_ and R′ = Me.

## Results and discussion

### Effect of the structure of the amine ligand on the efficiency of the catalytic system

**Fig. 3:**
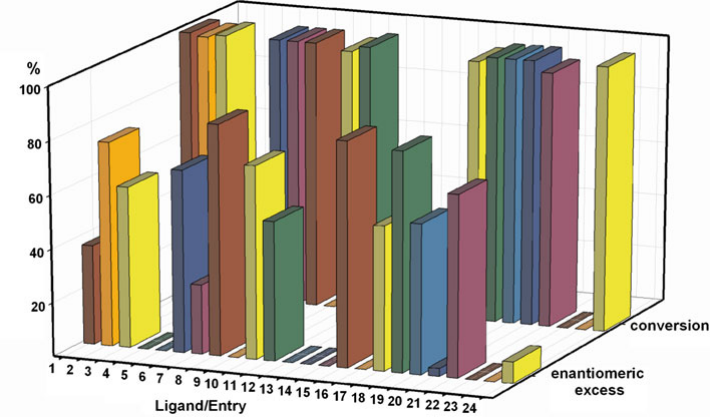
Effect of ligand structure on conversion and enantioselectivity of hydrosilylation of 4-methylacetophenone. Reaction conditions: 3.5 mol % catalyst, 1.2 equiv. silane, reaction time 24 h, toluene, RT; conversions and enantiomeric excesses were determined by HPLC with a Chiralpak IA column, average of two runs

We initially examined the effect of ligand structure on the yield and enantioselectivity of Zn-catalyzed hydrosilylation of 4-methylacetophenone (Fig. 3). The catalyst load in all cases was 3.5 mol %. Diphenylsilane was used as the reducing agent and all reactions were carried out in dry and degassed toluene for 24 h at room temperature.

Among 24 ligands tested nine (**L4**, **L5**, **L9**, **L12**–**L14**, **L16**, **L22**, and **L23**) did not promote hydrosilylation under these conditions (Fig. 3, entries 4, 5, 9, 12–14, 16, and 22–23), resulting in recovery of the starting material. These non-effective ligands contain either polar or bulky groups in different positions on the benzene ring (**L4**, **L5**, **L9**, **L16**) or have an additional substituent on the nitrogen atom (**L13**, **L14**, **L23**).

Ligands having additional hydroxy (**L4**) or methoxy groups (**L5**) in the *para* position of the benzene ring were inactive in the hydrosilylation reaction, in contrast with ligand **L6**, with dimethylamino groups in the *para* positions (Fig. 3, entry 6). It is interesting that the conformationally labile ligands **L7** and **L20** with hydroxy groups in the *ortho* positions which do not form planar complexes with zinc, are, nevertheless, active hydrosilylation catalysts, with conversions of the substrate almost quantitative, although with low enantiomeric excess of the product (Fig. 3, entries 7 and 20).

Catalytically inactive acyclic (**L13** and **L14**) and macrocyclic (**L23**) ligands are distinguished by additional methyl substituents on the nitrogen atoms. This indicates that the presence of an N–H bond in the ligand structure is essential if the reaction is to proceed. Macrocyclic ligand **L24**, characterized by the presence of hydroxyethyl substituents on the nitrogen atoms, is an exception. Although it activates the catalyst, the enantioselectivity obtained by use of **L24** was low and the best result was obtained when six equivalents of ZnEt_2_ per one equivalent of the ligand were used (ee 15 %, conversion of the substrate 99 %).

Although salen **L16** is catalytically inactive, apparently because of the formation of dimeric species [41] its methoxy derivative **L17** promotes the hydrosilylation reaction (Fig. 3, entry 17).

The presence of an N–H hydrogen atom is a prerequisite for catalytic activity; it is, however, not sufficient for catalysis of the hydrosilylation reaction. In secondary diamine **L12**, an aniline derivative, delocalization of the nitrogen lone pair to the aromatic ring reduces its coordination ability. More importantly, the specific, almost parallel conformation of the aromatic rings in **L12** does not allow the zinc atom to be included in the coordination space of the nitrogen atom [42]. This makes the ligand ineffective for catalysis.

In **L22** the phenyl groups on the benzylic carbon atoms occupy positions perpendicular to the average plane of the macrocycle [43, 44] and thus strongly stabilize the triangular conformation of **L22**. Steric crowding and lack of flexibility of the ligand apparently prohibit substrate binding. In contrast with **L22**, less sterically crowded **L21** catalyses the hydrosilylation reaction efficiently (Fig. 3, entry 21).

The enantiomeric excess of the product obtained by use of **L15** is the second best for all ligands tested; however, conversion of the substrate does not exceed 5 % (Fig. 3, entry 15). Therefore **L15** is not efficient as a ligand.

These results clearly show that both overcrowding and too much space around the coordination site make the efficiency of the catalytic system problematic. Ligands **L1** and **L2** differ in substitution of nitrogen atoms. Although both ligands are catalytically active in terms of conversion of the substrate (over 99 and 98 %, respectively for **L1** and **L2**; Fig. 3, entries 1 and 2), the enantioselectivity of the reaction with use of *N*-benzyl derivative **L2** is ca twice as high (76 %) as that obtained by use of the simple *N*-methyl DACH derivative **L1** (37 %) and may be further increased if the diamine is part of a regular trianglamine **L18** structure. In this case conversion of the substrate is quantitative and the macrocyclic structure of the ligand results in an increase in the enantioselectivity (82 %) of the process (Fig. 3, entry 18).

**Fig. 4:**
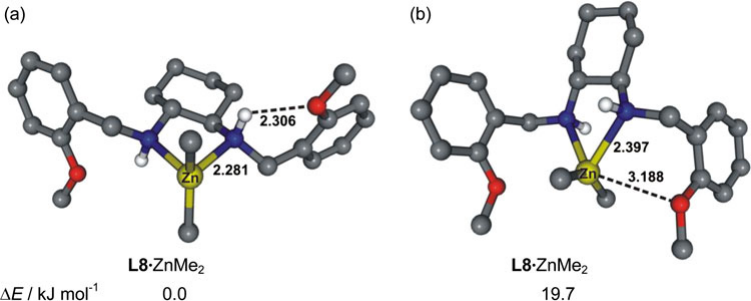
Structures of stable conformers **L8** ZnMe_2_ (**a**) and **L8** ZnMe_2_ (**b**) calculated at the PBE0/6-311++G(d,p) level of theory (some hydrogen atoms were omitted for clarity; distances are in Å)

Efficiency of the catalyst may be further tuned by introduction of additional coordinating groups, although with varying results. Replacement of the phenyl group by a 2-pyridyl group (**L11**) results in an increase of conversion at the expense of enantioselectivity (52 %), in comparison with **L2** (Fig. 3, entry 11). Introduction of a diphenylphosphine group in the ligand **L10** structure results in an increase of enantioselectivity (to 72 %; Fig. 3, entry 10), but enantioselectivity is still lower than with use of **L2**. The best result in terms of product enantioselectivity (86 %) and conversion of the substrate (>99 %) was obtained by use of methoxy-substituted ligand **L8** (Fig. 3, entry 8). Direct (Me)O···Zn interactions are excluded in the complex of **L8** and dialkylzinc. According to computation at the PBE0/6-311++G(d,p) level [45] the stable conformer **L8** ZnMe_2_ (Fig. 4b), in which a direct O···Zn interaction is possible, is of higher energy (19.7 kJ mol^−1^) than the conformer with an intramolecular NH···O(Me) hydrogen bond (Fig. 4).

Screening of the ligands was conducted in conjunction with screening of the type of silane used as the reducing agent. Selected results are shown in Table 1.

**Table 1:** Effect of silane structure on conversion and enantioselectivity of the hydrosilylation of 4-methylacetophenone

Entry	Silane	Ligand	Conversion/%	ee/%
1	PMHS	**L2**	>99	55
2	(EtO)_3_SiH	**L8**	>99	85
3	Ph_2_SiH_2_	**L8**	>99	86
4	PMHS	**L11**	>99	41
5	PMHS	**L18**	99	81
6	(EtO)_3_SiH	**L18**	>99	78
7	Et_3_SiH	**L18**	Traces	7
8	PhSiH_3_	**L18**	>99	78
9	Me_2_PhSiH	**L18**	0	0

3.5 mol % catalyst, 1.2 equiv. silane, reaction time 24 h, toluene, RT; conversion and enantiomeric excess were determined by HPLC with a Chiralpak IA column (average of two runs)

In the test reaction none of the silanes tested (PMHS, Et_3_SiH, (EtO)_3_SiH, Me_2_PhSiH, and PhSiH_3_) performed better than diphenylsilane in combination with **L8**. The decrease of enantioselectivity was small when (EtO)_3_SiH was combined with **L8** (drop to 85 %; Table 1, entry 2) or **L18** (drop to 81 %; Table 1, entry 4), compared with the combination of Ph_2_SiH_2_ and **L8**. In other cases, however, it was significant. Among silanes tested, two (Et_3_SiH, Me_2_PhSiH) did not promote hydrosilylation under the above mentioned conditions. In all cases discussed in this paragraph the product of hydrosilylation had *S* absolute configuration.

### Hydrosilylation of aryl–alkyl or aryl–aryl ketones catalyzed by [Zn(**L8**)] or [Zn(**L18**)] complexes

Having established **L8** and **L18** as the best performing chiral ligands, a variety of alkyl–aryl and aryl–aryl ketones were reduced by using 3.5 mol % catalyst, 1.2 equiv. silane, and toluene as solvent. The most interesting results are summarized in Table 2.

**Table 2:** [Zn(Diamine)]-catalyzed hydrosilylation of prochiral ketones

Entry	Substrate	Silane	Ligand	ee/% [yield/%]^a^
1	C_6_H_5_COC_2_H_5_	Ph_2_SiH_2_	**L8**	74 (*S*)^b^
2	C_6_H_5_COC_2_H_5_	Ph_2_SiH_2_	**L18**	85 (*S*)
3	C_6_H_5_COC_2_H_5_	PMHS	**L18**	86 [71] (*S*)
4	C_6_H_5_COC_6_H_11_	Ph_2_SiH_2_	**L8**	74 (*S*)
5	C_6_H_5_COC_6_H_11_	Ph_2_SiH_2_	**L18**	83 (*S*)
6	4-MeO–C_6_H_4_COCH_3_	Ph_2_SiH_2_	**L8**	79 [44] (*S*)
7	4-MeO–C_6_H_4_COCH_3_	Ph_2_SiH_2_	**L18**	43 [60] (*S*)
8	4-F–C_6_H_4_COCH_3_	PMHS	**L18**	78 (*S*)
9	4-F–C_6_H_4_COCH_3_	PhSiH_3_	**L18**	81 (*S*)
10	4-F–C_6_H_4_COCH_3_	Ph_2_SiH_2_	**L18**	82 [63] (*S*)
11	4-NC–C_6_H_4_COCH_3_	Ph_2_SiH_2_	**L8**	80 [26] (*S*)
12	4-NC–C_6_H_4_COCH_3_	PMHS	**L18**	71 (*S*)
13	4-NC–C_6_H_4_COCH_3_	PhSiH_3_	**L18**	69 (*S*)
14	Indanone	Ph_2_SiH_2_	**L8**	32 (*S*)
15	Indanone	Ph_2_SiH_2_	**L18**	69 [75] (*S*)
16	1-Tetralone	Ph_2_SiH_2_	**L8**	84 [91] (*S*)
17	1-Tetralone	Ph_2_SiH_2_	**L18**	71 [97] (*S*)
18	2-Tetralone	Ph_2_SiH_2_	**L8**	39 [75] (*S*)
19	2-Tetralone	Ph_2_SiH_2_	**L18**	12 [62] (*S*)
20	C_6_H_5_COCF_3_	Ph_2_SiH_2_	**L8**	15 [43] (*R*)
21	C_6_H_5_COCF_3_	Ph_2_SiH_2_	**L18**	19 [54] (*R*)
22	C_6_H_5_COCF_3_	PMHS	**L18**	11 [50] (*R*)
23	2,4,6-Me_3_C_6_H_2_COCF_3_	Ph_2_SiH_2_	**L8**	6 (*R*)
24	2,4,6-Me_3_C_6_H_2_COCF_3_	PMHS	**L18**	19 [52] (*R*)
25	4-F–C_6_H_4_COCF_3_	Ph_2_SiH_2_	**L8**	6 (*R*)
26	4-F–C_6_H_4_COCF_3_	PMHS	**L18**	9 [34] (*R*)
27	3,5-(CF_3_)_2_–C_6_H_3_COCH_3_	Ph_2_SiH_2_	**L8**	68 [76] (*S*)
28	3,5-(CF_3_)_2_–C_6_H_3_COCH_3_	PMHS	**L18**	16 [64] (*S*)
29	3-Me–C_6_H_4_COC_6_H_5_	Ph_2_SiH_2_	**L8**	13 [81] (*S*)
30	3-Me–C_6_H_4_COC_6_H_5_	Ph_2_SiH_2_	**L18**	1 (*S*)
31	4-Me–C_6_H_4_COC_6_H_5_	Ph_2_SiH_2_	**L8**	7 (*S*)
32	4-Me–C_6_H_4_COC_6_H_5_	Ph_2_SiH_2_	**L18**	11 [92] (*S*)
33	2-Cl–C_6_H_4_COC_6_H_5_	Ph_2_SiH_2_	**L8**	62 (*R*)
34	2-Cl–C_6_H_4_COC_6_H_5_	Ph_2_SiH_2_	**L18**	72 [84] (*R*)
35	4-Cl–C_6_H_4_COC_6_H_5_	Ph_2_SiH_2_	**L8**	26 (*R*)
36	4-Cl–C_6_H_4_COC_6_H_5_	Ph_2_SiH_2_	**L18**	26 [47] (*R*)

3.5 mol % catalyst, 1.2 equiv. silane, reaction time 24 h, toluene, RT; conversion and enantiomeric excess were determined by HPLC with a Chiralpak IA column (average of two runs)

^a^Isolated yield

^b^Absolute configuration

Of all substrates ethyl phenyl ketone gave the best results in terms of enantioselectivity and yield of the reaction (ee up to 86 %; Table 2, entries 2 and 3). The presence of a bulky alkyl group in cyclohexyl phenyl ketone resulted in a small decrease of enantioselectivity (83 %; Table 2, entry 5). For both compounds, use of macrocycle **L18** provided better results than the use of acyclic ligand **L8**.

Enantiomeric purity varied significantly when cyclic ketones, 1-tetralone, 2-tetralone, and 1-indanone, were used as the substrates. The best result was obtained in the reduction of 1-tetralone by diphenylsilane in the presence of ligand **L8** (ee 84 %, yield 91 %; Table 2, entry 16). Better yield (97 %) at the expense of enantioselectivity (drop to 71 %) was achieved when zinc complex with **L18** was used as the catalyst for reduction of 1-tetralone (Table 2, entry 17). The opposite trend was observed on use of 1-indanone—higher enantiomeric excess (69 %) was obtained by use of **L8** (Table 2, entry 15). 2-Tetralone was an exception—because of the low stereodifferentation of both faces of the substrate molecule, enantioselectivity of the reaction did not exceed 40 % when **L8** was used as ligand (Table 2, entry 18).

4-Methoxyacetophenone, 4-cyanoacetophenone, and 4-fluoroacetophenone gave the products of hydrosilylation with good ee (79–82 %; Table 2, entries 6–7 and 10–13).

Pharmaceutically important aryl trifluoromethyl ketones were hydrosilylated with low enantioselectivity (ee 19 %; Table 2, entries 21 and 24, and 9 %; Table 2, entry 26). The lack of success was in agreement with previously reported data [30]. On the other hand, trifluoromethyl groups attached to the aryl ring affected reaction enantioselectivity to a small extent only. When 3,5-bis(trifluoromethyl)phenyl methyl ketone was used the enantioselectivity of the reaction (up to 68 %) was over four times more than when phenyl trifluoromethyl ketone was used, and the product was obtained with good chemical yield (76 %; Table 2, entry 27) when **L8** was used as a ligand.

Hydrosilylation of substituted benzophenones gave mixed results (Table 2, entries 29–36). The best result (ee 72 %, yield 84 %) was obtained when 2-chlorobenzophenone was reduced chemoselectively by diphenylsilane with use of **L18** (Table 2, entry 34).

### Asymmetric activation of the [Zn(diamine)] catalyst

Use of an activator in the [Zn(diamine)]-catalyzed hydrosilylation reactions has the potential to increase both enantioselectivity and the reaction rate. In our study we tested several diol–amine combinations including acyclic ligands **L1**, **L2**, macrocycle **L18**, and diol activators **D1**–**D14** (Figs. 1, 2). Note that **L2** was used in either the racemic or enantiomerically pure form.

Standard conditions involved generation of the zinc complex by addition of an equimolar amount of diethylzinc in hexane to a solution of the ligand in toluene (or any other solvent) followed by addition of an equimolar amount of the appropriate diol. After 0.5 h the ketone and the silane were added and reaction was performed at room temperature for 24 h and then quenched with methanolic NaOH solution (Scheme 2). Changing of the order of addition of ZnEt_2_, diamine, activator, silane, and substrate did not affect enantioselectivity or conversion rate.

Although the nature of the silane had only a negligible effect on the conversion rate and enantioselectivity of the reaction, slightly better results were obtained when diphenylsilane was used as the reducing agent.

The effect of activator structure and the reaction conditions on the efficiency of the catalytic system was studied in the test reaction of hydrosilylation of 4-methylacetophenone. The results are summarized in Table 3.

**Table 3:** Effect of ligand structure and solvent on the efficiency of [Zn(diamine)(diol)]-catalyzed hydrosilylation of 4-methylacetophenone

Entry	Solvent	Ligand	Activator	Conversion/%	ee/%
1	Toluene	**L1**	**D1**	100	41
2	Toluene	**L2**	**D1**	98	75
3	Toluene	**L2**	**D1** ^a^	>99	78
4	Toluene	**L18**	**D1**	99	82
5	Toluene	**L1**	(*rac*)-**D9**	100	40
6	Toluene	**L18**	(*rac*)-**D9**	96	83
7	Toluene	**L1**	(*R*)-**D9**	100	41
8	Toluene	**L18**	(*R*)-**D9**	97	73
9	Toluene	**L18**	(*R*)-**D9** ^a^	92	73
10	Toluene	**L1**	(*S*)-**D9**	100	38
11	Toluene	**L18**	(*S*)-**D9**	>99	81
12	THF	**L18**	**D1**	>99	81
13	CH_2_Cl_2_	**L18**	**D1**	>99	69
14	Toluene	**L18**	MeOH^b^	98	79
15	Toluene	**L18**	MeOH^a^	>99	84
16	MeOH	**L18**	None	97	61
17	Toluene	(*rac*)-**L2**	(*R*)-**D2**	>99	4
18	Toluene	**L2**	(*rac*)-**D2**	99	77
19	Toluene	None	**D6**	0	0
20	Toluene	None	**D6** ^a^	0	0
21	Toluene	(*rac*)-**L2**	**D6**	>99	5
22	Toluene	**L2**	**D6**	>99	72
23	Toluene	None	**D7**	0	0
24	Toluene	(*rac*)-**L2**	**D7**	>99	6

3.5 mol % catalyst, 1.2 equiv. diphenylsilane, reaction time 24 h, toluene, RT; conversion and enantiomeric excess were determined by HPLC with a Chiralpak IA column (average of two runs)

^a^2 equiv. activator were used

^b^1 equiv. MeOH was used

The effect of amine and diol structure on the enantioselectivity of [Zn(diamine)(diol)]-catalyzed hydrosilylation was investigated for two arbitrarily chosen activators **D1** and **D9**. Enantioselectivity obtained for [Zn(diamine)**D1**]-catalyzed hydrosilylations ranged from 41 (**L1**) to 82 % (**L18**) and increased slightly (to 83 %) if 2 equiv. **D1** were used for 1 equiv. **L18** (Table 3, entries 1–4). The presence of the stereogenic center in **D9** did not significantly affect the stereoselectivity of the reaction. Comparison of the results obtained for Zn(**L1**) and Zn(**L18**) complexes activated by either (*rac*), (*R*), or (*S*)-**D9** shows the advantage of cyclic over acyclic structure of the amine ligand and in each series similar results were obtained when either (*rac*)-**D9** or enantiomerically pure **D9** was used as the activator (Table 3, entries 5–11). For [Zn(**L18**)(**D9**)]-catalyzed hydrosilylations of 4-methylacetophenone, for example, product enantioselectivity was: 83, 73, and 81 % for (*rac*), (*R*), and (*S*)-**D9**, respectively. The enantioselectivity of the non-activated [Zn(**L18**)]-catalyzed reaction was 82 %.

Changing the solvent from toluene to THF or dichloromethane slightly improved conversion in the [Zn(**L18**)(**D1**)]-catalyzed hydrosilylation of 4-methylacetophenone, from 98 to 100 %, at the expense of enantioselectivity (drop to 81 or 69 % ee of the product; Table 3, entries 12 and 13). Substituting the diol in the catalyst structure with 2 or 1 equiv. methanol resulted in a 1 and 6 % (respectively for 2 or 1 equiv. MeOH) drop in enantioselectivity of the hydrosilylation reaction performed in toluene (Table 3, entries 14 and 15). The same reaction could be performed in pure methanol (Table 3, entry 16). Although conversion of the substrate was still high, enantiomeric purity of the product decreased substantially (drop to 61 %). In contrast with the reduction of ketones, a similar reaction performed for imines gave better results in protic solutions than in any non-polar or aprotic media [31].

The stereochemical course of the reaction is controlled by the chirality of the diamine part of catalytically active species. Although use of (*rac*)-**L2** with enantiomerically pure diols **D2**, **D6**, and **D7** preserved high conversion of the substrate, we observed a substantial decrease of enantioselectivity (Table 3, entries 17, 21, and 24). Use of enantiomerically pure **L2** with (*rac*)-**D2** as the catalytic system provided the product with 77 % ee (Table 3, entry 18). It should be noted that when used alone, the diol or [Zn(diol)] were not catalytically active species (Table 3, entries 19, 20, and 23).

**Fig. 5:**
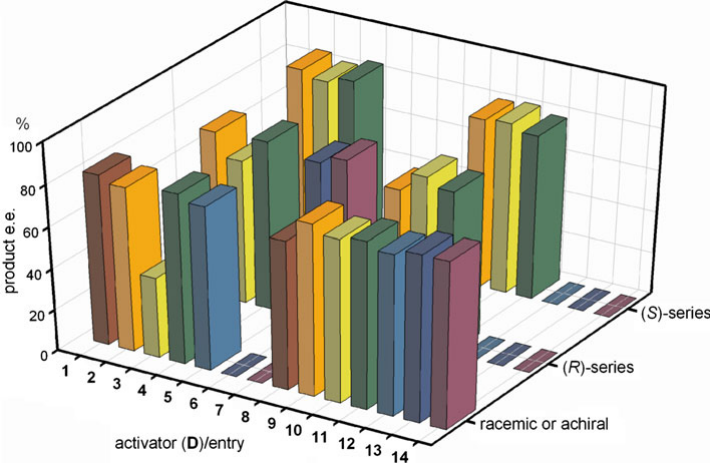
Effect of activator structure on enantioselectivity in [Zn(**L18**)]-catalyzed hydrosilylation of 4-methylacetophenone. First row represents achiral or racemic activators, second and third rows summarize results obtained for activators of *R* or *S* absolute configuration at the stereogenic centers, respectively

Full screening of the activators was conducted for [Zn(**L18**)]-catalyzed hydrosilylation of 4-methylacetophenone. All reaction were carried out in toluene on a 1 mmol scale, using 3.5 mol % **L18**, equimolar amounts of ZnEt_2_ and the activator, and 1.2 mmol silane. Conversion and enantiomeric excess were determined by HPLC with a Chiralpak IA column. The results are summarized in Fig. 5.

The results show that all combinations of **L18** and the activator were catalytically active. As was mentioned above, conversion of the substrate was high (over 92 %) and enantiomeric excess of the product was usually ca. 80 %.

More detailed inspection of the results summarized in Fig. 5 shows that the highest enantioselectivity (ee 85 %) in the hydrosilylation of 4-methylacetophenone was achieved with trianglamine ligand **L18** and (*S*)-**D2** or (*S*,*S*)-**D4** as activator, with diphenylsilane as reducing agent (Fig. 5, entries 2 and 4). Replacing (*S*)-**D2** or (*S*,*S*)-**D4** by the simple diol **D1** resulted in a 3 % drop of enantioselectivity, with unchanged conversion (Table 3, entry 4). Replacing the aliphatic alcohol by phenol as activator had no profound effect on either enantioselectivity or conversion of the substrate. The product of hydrosilylation had *S* absolute configuration irrespective of the amine–alcohol combination used.

## Summary

In this work we tested a wide range of DACH-based amine ligands, activators, and reactants for asymmetric hydrosilylation of prochiral ketones. The **L8**-ZnEt_2_ and **L18**-ZnEt_2_ (1:1) complexes catalyze the hydrosilylation of 4-methylacetophenone by diphenylsilane in toluene solution, with up to 86 % ee of the product. The enantioselectivity is higher than that with use of other acyclic or cyclic ligands. Because both **L8** and **L18** can be conveniently prepared by a one-pot procedure from the inexpensive tartrate salt of DACH and *o*-anisole or terephthalaldehyde, the Zn complexes of **L8** and **L18** are good alternatives to previously reported chiral catalysts.

A small effect of activator structure on enantioselectivity and significant large increases in the conversion and yield of the reaction seem consistent with the mechanism postulated by Bette et al. [39] and by us, supported by computational experiments [46]. According to this proposal the diol moiety does not sterically affect the substrate in the transition state; it does, however, activate the zinc ion acting as catalyst [39, 40].

## Experimental

NMR spectra were recorded on Bruker BioSpin 400 (400 MHz) or Varian MR 300 (300 MHz) instruments at 25 °C using CDCl_3_ as solvent. Chemical shifts are reported in ppm relative to the TMS peak (^1^H and ^13^C NMR spectra). Spectral assignments were obtained by analysis of chemical shifts and by comparison with literature data. Mass spectra were recorded on an AMD-402 spectrometer. HPLC analysis was performed at room temperature with an Hitachi LaChrom Elite system equipped with a Chiralpak IA column. Details of HPLC separation of the enantiomers are given in the Supplementary Information (Table SI1). Absolute configurations of the products were determined by comparison of measured optical rotation with the literature data [47–62].

Ligands **L1**–**L3**, **L5**, **L7**–**L18**, **L21**–**L24** were prepared by procedures reported in the literature [24–30, 43, 63–71]. Ligands **L4**, **L4**, **L19**, and **L20** were obtained from the corresponding imines [28, 65, 72], by use of a procedure reported elsewhere [24]. NMR spectra and melting points for all ligands and products of hydrosilylation reactions were in agreement with literature data (systematic names of **L1**–**L3**, **L5**, **L7**–**L18**, **L21**–**L24** and some physical data are given in the Supplementary Information, Table SI2) [24–30, 41, 43, 47–71]. Diols **D1**–**D14** were commercial products.

### *(R,R)*-*N,N′*-*Bis[(4*-*hydroxyphenyl)methyl]*-*1,2*-*cyclohexanediamine* (**L4**, C_20_H_26_N_2_O_2_)

Yield 86 %; white solid; m.p.: 141–144 °C (ethyl acetate–hexane); ^1^H NMR (400 MHz, CDCl_3_): *δ* = 7.11–7.08 (m, 4H), 6.79–6.72 (m, 4H), 3.79 (d, *J* = 12.7 Hz, 2H), 3.51 (d, *J* = 12.8 Hz, 2H), 3.27 (s, 2H), 2.26–2.24 (m, 2H), 2.17–2.13 (m, 2H), 2.06 (s, 2H), 1.75–1.73 (m, 2H), 1.42–1.35 (m, 4H) ppm; ^13^C NMR (101 MHz, CD_3_OD): *δ* = 158.3, 131.1, 129.7, 116.5, 60.5, 50.0, 30.7, 25.8 ppm; IR (KBr): $\bar{v}$ = 3,368, 3,295, 3,234, 2,940, 1,610, 1,598, 1,516, 1,247, 996, 829 cm^−1^; HR-EI-MS (*m*/*z*): [M]^+^ calcd for C_20_H_26_N_2_O_2_ 326.4326, found 326.4350.

### *(R,R)*-*N,N’*-*Bis[(4*-*dimethylaminophenyl)methyl]*-*1,2*-*cyclohexanediamine* (**L6**, C_24_H_36_N_4_)

Yield 83 %; white solid; m.p.: 140–146 °C (ethyl acetate–hexane); ^1^H NMR (300 MHz, CDCl_3_): *δ* = 7.12–6.97 (m, 4H), 6.65–6.50 (m, 4H), 3.90–3.86 (m, 2H), 3.85–3.81 (m, 2H), 2.95–2.91 (s, 12H), 2.43–2.39 (m, 2H), 1.92–1.85 (m, 2H), 1.84–1.80 (m, 2H), 1.69–1.65 (m, 2H), 1.38–1.34 (m, 2H), 1.33–1.29 (m, 2H) ppm; ^13^C NMR (75 MHz, CDCl_3_): *δ* = 160.7, 151.7, 129.2, 124.9, 111.5, 73.9, 40.2, 33.4, 24.7 ppm; IR (KBr): $\bar{v}$ = 3,430, 2,920, 2,852, 2,826, 1,633, 1,610, 1,528, 1,370, 1,181, 807 cm^−1^; HR-EI-MS (*m*/*z*): [M]^+^ calcd for C_24_H_36_N_4_ 380.5694, found 380.5701.

### *(2R,3R,16R,17R,30R,31R)*-*1,4,15,18,29,32*-*Hexaaza*-*(2,3:16,17:30,31)*-*tributano*-*(8,11:22,25:36,39)*-*trietheno*-*1,2,3,4,5,14,15,16,17,18,19,28,29,30,31,32,33,42*-*octadecahydro[42]annulene* (**L19**, C_54_H_72_N_6_)

Yield 84 %; white solid: m.p.: >320 °C (dichloromethane–hexane); ^1^H NMR (300 MHz, CDCl_3_): *δ* = 7.25 (s, 6H), 6.53 (d, *J* = 15.2 Hz, 6H), 6.22–6.16 (m, 6H), 3.29–3.25 (m, 6H), 3.19–3.15 (m, 6H), 2.55–2.49 (m, 6H), 1.72 (s, 6H), 1.69–1.61 (m, 6H), 1.52–1.45 (m, 6H), 1.44–1.34 (m, 12H) ppm; ^13^C NMR (126 MHz, CDCl_3_): *δ* = 136.3, 130.5, 128.8, 126.4, 126.1, 61.1, 49.1, 49.0, 31.4, 25.0 ppm; IR (KBr): $\bar{v}$ = 3,424, 3,296, 2,928, 2,854, 2,370, 1,757, 1,450, 1,238, 1,120, 1,050, 964, 750 cm^−1^; HR-EI-MS (*m*/*z*): [M]^+^ calcd for C_54_H_72_N_6_ 805.1897, found 805.1905.

### *(2R,3R,11R,12R,20R,21R)*-*1,4,10,13,19,22*-*Hexaaza*-*(2,3:11,12:20,21)*-*tris(tetramethylene)*-*7,16,25*-*trihydroxy*-*(6,8:15,17:24,26)*-*tris(2*-*tert*-*butyl*-*1*-*propen*-*3*-*yl)*-*1,2,3,4,5,9,10,11,12,13,14,18,19,20,21,22,23,27*-*octadecahydro[27]annulene* (**L20**, C_54_H_84_N_6_O_3_)

Yield 93 %; glass; ^1^H NMR (400 MHz, CDCl_3_): *δ* = 7.09 (s, 6H), 5.05 (s, 3H), 3.81 (m, 12H), 2.53–2.51 (m, 6H), 1.96 (s, 6H), 1.69–1.61 (m, 6H), 1.53–1.45 (m, 6H), 1.44–1.34 (m, 39H) ppm; ^13^C NMR (75 MHz, CDCl_3_): *δ* = 154.5, 154.0, 140.8, 125.0, 123.9, 60.3, 48.8, 48.2, 33.8, 31.6, 31.4, 30.8, 24.8 ppm; IR (KBr): $\bar{v}$ = 3,269, 2,950, 2,858, 1,609, 1,484, 1,362, 1,215 cm^−1^; HR-EI-MS (*m*/*z*): [M]^+^ calcd for C_54_H_84_N_6_O_3_ 865.6605, found 865.6596.

### General procedure for Zn(diamine)]-catalyzed hydrosilylation of ketones

In a 5-cm^3^ round-bottomed flask 12.5 mm^3^ ZnEt_2_ (1 M in hexane, 0.0125 mmol) and the appropriate chiral ligand (0.0125 mmol) were dissolved in 1.5 cm^3^ freshly distilled toluene and stirred under an argon atmosphere for 30 min. The corresponding ketone (0.344 mmol), or its solution in 1 cm^3^ toluene, and the silane (0.413 mmol) were then added to the mixture. The resulting solution was stirred at room temperature for 24 h. NaOH (1 M in MeOH; 1 cm^3^) was then added with vigorous stirring. The reaction mixture was stirred for an additional hour at room temperature and then the solvents were evaporated. The residue was dissolved in a mixture of 10 cm^3^ H_2_O and 1 cm^3^ 10 % HCl and extracted with diethyl ether (3 × 10 cm^3^). The combined organic extracts were washed with saturated aqueous NaHCO_3_ solution, H_2_O, and brine, dried over anhydrous MgSO_4_, and concentrated under vacuum. The product was purified by column chromatography on silica gel with hexane–EtOAc (10:1) as eluent.

### General procedure for Zn(diamine)(diol)]-catalyzed hydrosilylation of 4-methylacetophenone

In a 5-cm^3^ round-bottomed flask 12.5 mm^3^ ZnEt_2_ (1 M in hexane, 0.0125 mmol), the appropriate chiral ligand (0.0125 mmol), and the diol (0.0125 mmol) were dissolved in 1.5 cm^3^ freshly distilled toluene and stirred under an argon atmosphere for 30 min. 4-Methylacetophenone (0.344 mmol) and silane (0.413 mmol) were then added to the mixture. The resulting solution was stirred at room temperature for 24 h, then 1 cm^3^ NaOH (1 M in MeOH) was added with vigorous stirring. The reaction mixture was stirred for an additional hour at room temperature and the solvents were then evaporated. The residue was dissolved in a mixture of 10 cm^3^ H_2_O and 1 cm^3^ 10 % HCl and extracted with diethyl ether (3 × 10 cm^3^). The combined organic extracts were washed with saturated aqueous NaHCO_3_ solution, H_2_O, and brine, dried over anhydrous MgSO_4_, and concentrated under vacuum. The product was purified by column chromatography on silica gel with hexane–EtOAc (10:1) as eluent.

## Electronic supplementary material

Below is the link to the electronic supplementary material.

Supplementary material 1 (PDF 328 kb)
