# Supplementary material for: Asymmetric hydrosilylation of ketones catalyzed by complexes formed from trans-diaminocyclohexane-based diamines and diethylzinc
Source: Monatsh Chem. 2012 Apr 18;143(7):1045–54. doi: 10.1007/s00706-012-0754-0 (PMC4494764; doi:10.1007/s00706-012-0754-0)
Supplement: Supplementary file 1 — Supplementary material 1 (PDF 328 kb) [file 706_2012_754_MOESM1_ESM.pdf]

Supplementary Information for:

Asymmetric Hydrosilylation of Ketones Catalyzed by Complexes Formed from *trans*-Diaminocyclohexane-based Diamines and Diethylzinc

Jadwiga Gajewy ● Jacek Gawronski ● Marcin Kwit

Table SI1. Conditions and retention times for HPLC separation of enantiomers of ArCH(OH)R alcohols using a Chiralpak IA column.

Ar	R	Flow [ml·min^{-1}]	Hexane [%]	2-Propanol [%]	Retention time [min]	
					Minor	Major
Ph	Me	0.5	95	5	23.12	23.87
Ph	Et	0.5	95	5	16.76	17.29
Ph	Cy	0.5	95	5	22.29	20.95
4-Me-C_6H_4	Me	0.5	95	5	18.61	19.89
4-MeO-C_6H_4	Me	0.5	90	10	16.44	17.32
4-CN-C_6H_4	Me	0.55	90	10	16.75	17.36
4-F-C_6H_4	Me	1.0	99	1	21.97	22.87
3,5-CF_3-C_6H_3	Me	0.55	90	10	13.48	15.57
Ph	CF_3	0.5	95	5	19.89	21.13
4-F-C_6H_4	CF_3	0.5	95	5	18.35	19.91
2,4,6-$Me_3C_6H_2$	CF_3	0.5	95	5	15.41	13.81
3-Me-C_6H_4	Ph	0.55	99	1	55.00	51.79
4-Me-C_6H_4	Ph	0.5	95	5	18.24	19.49
2-Cl-C_6H_4	Ph	0.5	95	5	31.97	33.72
4-Cl-C_6H_4	Ph	0.5	95	5	44.83	41.28
1-Indanol		0.5	90	10	26.45	24.35

α-Tetralol	0.5	90	10	26.89	24.09
β-Tetralol	0.5	90	10	28.45	32.05

Table SI2. Systematic names, melting points and references to the NMR data for ligands **L1-L23**.

Ligand	Melting point and reference to the NMR data
**L1** 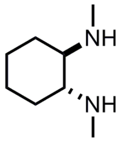	(*R*,*R*)-*N*,*N'*-Dimethyl-1,2-diaminocyclohexane<br>Commercial product;<br>E.-K. Lee, S.-H. Kim, B.-H. Jung, W.-S. Ahn, G.-J. Kim,<br>*Tetrahedron Letters***2003**, *44*, 1971.
**L2** 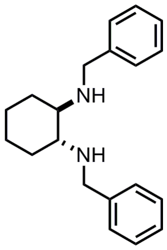	(*R*,*R*)-*N*,*N'*-Dibenzyl-1,2-diaminocyclohexane<br>Oil;<br>E.-K. Lee, S.-H. Kim, B.-H. Jung, W.-S. Ahn, G.-J. Kim,<br>*Tetrahedron Letters* **2003**, *44*, 1971.
**L3** 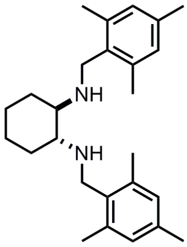	(*R*,*R*)-*N*,*N'*-Bis[(2,4,6-trimethylphenyl)methyl]-1,2-cyclohexanediamine<br>White solid, mp: 164 °C;<br>J. Etxebarria, H. Degenbeck, A.-S. Felten, S. Serres,<br>N. Nieto, A. Vidal-Ferran,<br>*J. Org. Chem.* **2009**,*74*, 8794.
**L5** 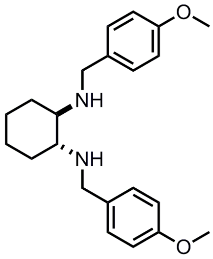	(*R*,*R*)-*N*,*N'*-Bis[(4-methoxyphenyl)methyl]-1,2-cyclohexanediamine<br>Oil;<br>T. Kylmälä, N. Kuuloja, Y. Xu, K. Rissanen, R. Franzén,<br>*Eur. J. Org. Chem.* **2008**, 4019.
**L7** 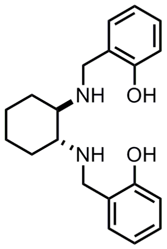	(*R*,*R*)-*N*,*N*'-Bis(salicyl)cyclohexane-1,2-diamine<br>Oil;<br>J. Sun, C. Zhu, Z. Dai, M. Yang, Y. Pan, H. Hu,<br>*J. Org. Chem.* **2004**, *69*, 8500.

**L8**	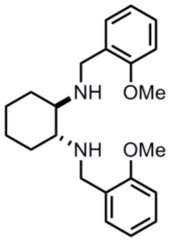	(*R*,*R)*-*N*,*N'*-Bis[(2-methoxyphenyl)methyl]-1,2-cyclohexanediamine<br>Oil;<br>T. Kylmälä, N. Kuuloja, Y. Xu, K. Rissanen, R. Franzén,<br>*Eur. J. Org. Chem.* **2008**, 4019.
**L9**	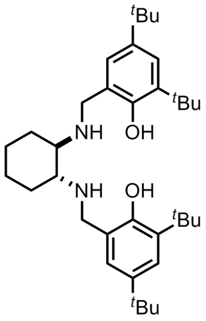	(*R*,*R*)-*N*,*N'*-Bis(3,5-di-*tert*-butylsalicyl)cyclohexane-1,2-diamine<br>White solid, m.p. 140-141 ˚C;<br>J. Sun, C. Zhu, Z. Dai, M. Yang, Y. Pan, H. Hu,<br>*J. Org. Chem.* **2004**, *69*, 8500.
**L10**	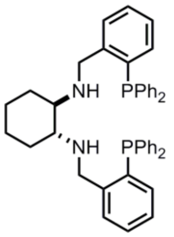	(*R*,*R*)-*N*,*N*'-Bis[*o*-(diphenylphosphino)benzyl]cyclohexane-1,2-diamine<br>White solid, m.p. 53-55 °C;<br>J.-X.Gao, X.-D. Yi, P.-P.Xu, C.-L. Tang, H.-L. Wan,<br>T.Ikariya,<br>*J. Organometal. Chem.* **1999**, *592*, 290.
**L11**	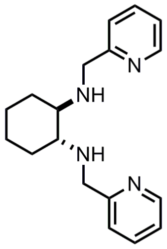	*N*,*N'*-Bis(2-pyridylmethyl)-(1*R*,2*R*)-cyclohexane-1,2-diamine<br>Oil;<br>W. Park, M. H. Shin, J. H. Chung, J. Park, M.S. Lahc,<br>D. Lim,<br>*Tetrahedron Letters* **2006**, *47*, 8841.
**L12**	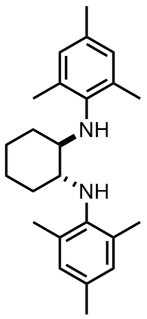	(*R*,*R*)-*N*,*N'*-Dimesityl-1,2-cyclohexanediamine<br>Yellow solid, m.p. 122 °C;<br>T. J. Seiders, D. W. Ward, R. H. Grubbs,<br>*Org. Lett.* **2001**, *3*, 3225.

**L13**	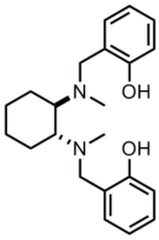	(*R*,*R*)-*N*,*N*’-Dimethyl-*N*,*N’*-bis(salicyl)cyclohexane-1,2-diamine<br>White solid, m.p. 119-120 °C;<br>J. Sun, C. Zhu, Z. Dai, M. Yang, Y. Pan, H. Hu,<br>*J. Org. Chem.* **2004**, *69,* 8500.
**L14**	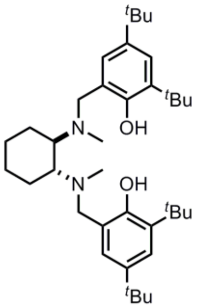	(*R*,*R*)-*N*,*N’*-Dimethyl-*N*,*N’*-bis(3,5-di-*tert*-butylsalicyl)cyclohexane-1,2-diamine<br>White solid, m.p. 100 °C;<br>J. Balsells, P. J. Carroll, P. J. Walsh,<br>*Inorg. Chem.* **2001,** *40*, 5568.
**L15**	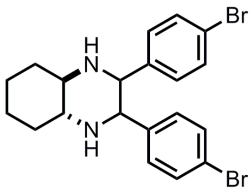	(4*αR*,8*αR*)-2,3-Bis(4-bromophenyl)decahydroquinoxaline<br>Yellow solid, m.p. 149-150 °C;<br>P. Hesemann, J. J. E. Moreau, T. Soto,<br>*Synth. Commun.* **2003**, *33*, 183.
**L16**	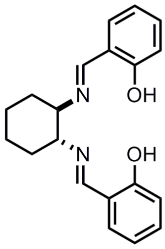	(*R*,*R*)-*N*,*N'*-Bis(salicylidene)-1,2-cyclohexanediamine<br>White solid, m.p. 118-119 °C;<br>T. Kylmälä, N. Kuuloja, Y. Xu, K. Rissanen, R. Franzén,<br>*Eur. J. Org. Chem.* **2008**, 4019.
**L17**	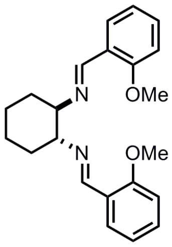	(*R,R)-N*,*N'*-Bis[(2-methoxyphenyl)methylene]-1,2-cyclohexanediamine<br>Yellowish solid; m.p. 107-108 °C;<br>T. Kylmälä, N. Kuuloja, Y. Xu, K. Rissanen, R. Franzén,<br>*Eur. J. Org. Chem.* **2008**, 4019.
**L18**	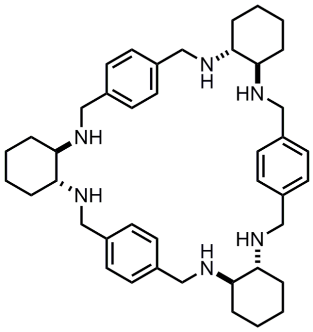	(2*R*,3*R*,12*R*,13*R*,22*R*,23*R*)-1,4,11,14,21,24-Hexa-aza-(2,3:12,13:22,23)-tributano-(6,9:16,19:26,29)-trietheno-(1*H*,2*H*,3*H*,4*H*,5*H*,10*H*,11*H*,12*H*,13*H*,14*H*,15*H*,20*H*,21*H*,22*H*,23*H*,24*H*,25*H*,30*H*)-octadecahydro-(30)-annulene<br>White solid, m.p. 154–156 °C;<br>J. Gawroński, K. Gawrońska, J. Grajewski, M. Kwit,

		A.Plutecka, U. Rychlewska,<br>*Chem. Eur. J.* **2006**, *12*, 1807.
**L21**	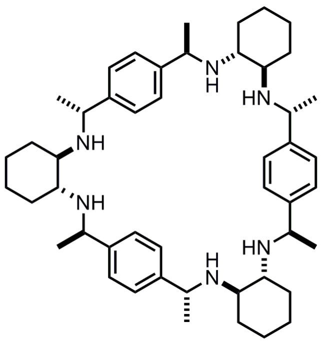	(2*R*,3*R*,5*R*,10*R*,12*R*,13*R*,15*R*,20*R*,22*R*,23*R*,25*R*,30*R*)-1,4,11,14,21,24-Hexa-aza-(2,3:12,13:22,23)-tributano-(6,9:16,19:26,29)-triettheno-(1*H*,2*H*,3*H*,4*H*,11*H*,12*H*,13*H*,14*H*,21*H*,22*H*,23*H*,24*H*)-dodecahydro-(5,10,15,20,25,30)-hexamethyl-(30)-annulene<br>White solid, m.p. 87–88 ˚C;<br>D. Savoia, A. Gualandia, H. Stoeckli-Evans,<br>*Org. Biomol. Chem.* **2010**, *8*, 3992.
**L22**	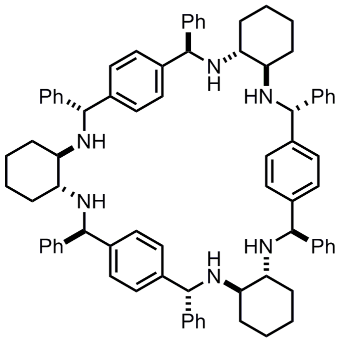	(2*R*,3*R*,5*R*,10*R*,12*R*,13*R*,15*R*,20*R*,22*R*,23*R*,25*R*,30*R*)-1,4,11,14,21,24-Hexa-aza-(2,3:12,13:22,23)-tributano-(6,9:16,19:26,29)-triettheno-(1*H*,2*H*,3*H*,4*H*,11*H*,12*H*,13*H*,14*H*,21*H*,22*H*,23*H*,24*H*)-dodecahydro-(5,10,15,20,25,30)-hexaphenyl-(30)-annulene<br>White crystals, m.p. 136–137 ˚C;<br>D. Savoia, A. Gualandia, H. Stoeckli-Evans,<br>*Org. Biomol. Chem.* **2010**, *8*, 3992.
**L23**	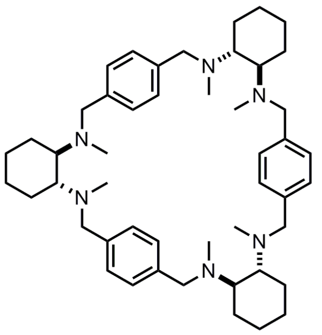	(2*R*,3*R*,12*R*,13*R*,22*R*,23*R*)-1,4,11,14,21,24-Hexa-aza-1,4,11,14,21,24-hexamethyl-(2,3:12,13:22,23)-tributano-(6,9:16,19:26,29)-triettheno-(1*H*,2*H*,3*H*,4*H*,5*H*,10*H*,11*H*,12*H*,13*H*,14*H*,15*H*,20*H*,21*H*,22*H*,23*H*,24*H*,25*H*,30*H*)-octadecahydro-(30)-annulene<br>White solid, m.p. 182–186 °C;<br>J. Gawroński, K. Gawrońska, J. Grajewski, M. Kwit,<br>A. Plutecka, U. Rychlewska,<br>*Chem. Eur. J.* **2006**, *12*, 1807.
**L24**	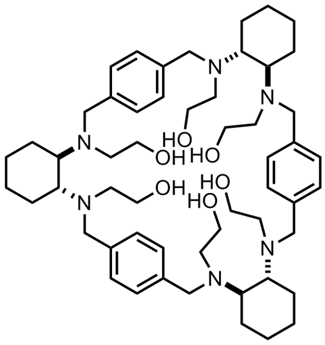	(2*R*,3*R*,12*R*,13*R*,22*R*,23*R*)-1,4,11,14,21,24-Hexa-aza-1,4,11,14,21,24-hexa(2-hydroxyethyl)-(2,3:12,13:22,23)-tributano-(6,9:16,19:26,29)-triettheno-(1*H*,2*H*,3*H*,4*H*,5*H*,10*H*,11*H*,12*H*,13*H*,14*H*,15*H*,20*H*,21*H*,22*H*,23*H*,24*H*,25*H*,30*H*)-octadecahydro-(30)-annulene<br>Glassy product;<br>J. Gawroński, K. Gawrońska, J. Grajewski, M.

	Kwit,<br>A.Plutecka, U. Rychlewska,<br>*Chem. Eur. J.* **2006**, *12*, 1807.

Table SI3. Systematic names, melting points and references to the NMR data for the products of hydrosilylation reactions.

Systematic name	Known compound	Melting point [°C]	Reference to melting point in literature	Reference to NMR data in literature
(*S*)-1-Phenylethanol	Yes	oil	–	1
(*S*)-1-Phenylpropan-1-ol	Yes	oil	–	1
(*S*)-Cyclohexylphenyl-methanol	Yes	48-50	2	1
(*S*)-1-(4-Methylphenyl)-ethanol	Yes	oil	–	3
(*S*)-1-(4-Methoxyphenyl)-ethanol	Yes	oil	–	1
(*S*)-1-(4-Cyanophenyl)-ethanol	Yes	oil	–	4
(*S*)-1-(4-Fluorophenyl)-ethanol	Yes	oil	–	3
(*S*)-1-[3,5-Bis(trifluoromethyl)phenyl]-ethanol	Yes	oil	–	5
(*R*)-2,2,2-Trifluoro-1-phenylethanol	Yes	oil	–	6
(*R*)-2,2,2-Trifluoro-1-(4-fluoro-phenyl)-ethanol	Yes	oil	–	7
(*R*)-2,2,2-Trifluoro-1-(2,4,6-trimethylphenyl)-ethanol	Yes	oil	–	6
(*S*)-(3-Methylphenyl)phenyl-methanol	Yes	51-52	8	8
(*S*)-(4-Methylphenyl)phenyl-methanol	Yes	59-60	9	9
(*S*)-(2-Chlorophenyl)phenyl-methanol	Yes	oil	9	9
(*S*)-(4-Chlorophenyl)phenyl-methanol	Yes	54-55	9	9
(*S*)-1-Indanol	Yes	71-72	10a	10b
(*S*)-α-Tetralol	Yes	oil	–	11
(*S*)-β-Tetralol	Yes	oil	–	11

Reference

1. Morris DJ, Hayes AM, Wills M (2006) J Org Chem 71:7035
2. Belén Díaz-Valenzuela M, Phillips SD, France MB, Gunn ME, Clarke ML (2009) Chem Eur J 15:1227.
3. Utsukihara T, Misumi O, Kato N, Kuroiwa T, Horiuchi CA (2006) Tetrahedron Asymmetry 17:1179
4. Shuanglong L; Christian W (2007) Org Lett 9:2965
5. Singh RP, Twamley B, Fabry-Asztalos L, Matteson DS, Shreeve JM (2000) J Org Chem 65:8123
6. Yong KH, Chong JM (2002) Org Lett 4:4139
7. Xu Q, Zhou H, Geng X, Chen P (2009) Tetrahedron 65:2232
8. Stanchev S, Rakovska R, Berova N, Snatzke G (1995) Tetrahedron Asymmetry 6:183
9. Wu X, Liu X, Zhao G (2005) Tetrahedron Asymmetry 16:2299
10. a) Siden T, Gerard J (1980) J Organomet Chem 197:199; b) Aupoix A, Bournaud C, Vo-Thanh G (2011) Eur J Org Chem 15:2772
11. Palmer M, Walsgrove T, Wills M (1997) J Org Chem 62:5226
